# Sindbis virus polyarthritis outbreak signalled by virus prevalence in the mosquito vectors

**DOI:** 10.1371/journal.pntd.0007702

**Published:** 2019-08-29

**Authors:** Jan O. Lundström, Jenny C. Hesson, Martina L. Schäfer, Örjan Östman, Torsten Semmler, Michaël Bekaert, Manfred Weidmann, Åke Lundkvist, Martin Pfeffer

**Affiliations:** 1 Department of Medical Biochemistry and Microbiology/Zoonosis Science Center, Uppsala University, Uppsala, Sweden; 2 Biological Mosquito Control, Nedre Dalälven Utvecklings AB, Gysinge, Sweden; 3 Department of Epidemiology and Population Health, Institute of Infection and Global Health, University of Liverpool, Liverpool, United Kingdom; 4 Department of Aquatic Resources, Institute of Coastal Research, Swedish University of Agricultural Sciences, Öregrund, Sweden; 5 Robert Koch Institute, NG 1 – Microbial Genomics, Berlin, Germany; 6 Institute of Aquaculture, University of Stirling, Stirling, United Kingdom; 7 Department of Medical Sciences, Uppsala University and Laboratory of Clinical Microbiology, Uppsala University Hospital, Uppsala, Sweden; 8 Institute of Animal Hygiene and Veterinary Public Health, University of Leipzig, Leipzig, Germany; The Pennsylvania State University, UNITED STATES

## Abstract

Polyarthritis and rash caused by Sindbis virus (SINV), was first recognised in northern Europe about 50 years ago and is known as Ockelbo disease in Sweden and Pogosta disease in Finland. This mosquito-borne virus occurs mainly in tropical and sub-tropical countries, and in northern Europe it is suggested to cause regularly reoccurring outbreaks. Here a seven-year cycle of SINV outbreaks has been referred to in scientific papers, although the hypothesis is based solely on reported human cases. In the search for a more objective outbreak signal, we evaluated mosquito abundance and SINV prevalence in vector mosquitoes from an endemic area in central Sweden. Vector mosquitoes collected in the River Dalälven floodplains during the years before, during, and after the hypothesised 2002 outbreak year were assayed for virus on cell culture. Obtained isolates were partially sequenced, and the nucleotide sequences analysed using Bayesian maximum clade credibility and median joining network analysis. Only one SINV strain was recovered in 2001, and 4 strains in 2003, while 15 strains were recovered in 2002 with significantly increased infection rates in both the enzootic and the bridge-vectors. In 2002, the Maximum Likelihood Estimated infection rates were 10.0/1000 in the enzootic vectors *Culex torrentium/pipiens*, and 0.62/1000 in the bridge-vector *Aedes cinereus*, compared to 4.9/1000 and 0.0/1000 in 2001 and 0.0/1000 and 0.32/1000 in 2003 Sequence analysis showed that all isolates belonged to the SINV genotype I (SINV-I). The genetic analysis revealed local maintenance of four SINV-I clades in the River Dalälven floodplains over the years. Our findings suggest that increased SINV-I prevalence in vector mosquitoes constitutes the most valuable outbreak marker for further scrutinising the hypothesized seven-year cycle of SINV-I outbreaks and the mechanisms behind.

## Author summary

The mosquito-borne Sindbis virus (SINV) causes polyarthritis and rash known as Ockelbo disease in Sweden and Pogosta disease in Finland. This mainly tropical and sub-tropical virus occurs in many countries, and in northern Europe it is suggested to cause reoccurring outbreaks every seventh year. The seven-year SIN outbreak cycle is commonly referred to in scientific papers, although the hypothesis is based solely on reported clinical cases. In the search for a more objective outbreak risk signal, we evaluated abundance and SINV prevalence in vector mosquitoes from Sweden. Vector mosquitoes collected in the River Dalälven floodplains the years before, during and after the hypothesized 2002 outbreak, were assayed for SINV. SINV prevalence was significantly increased in vector mosquitoes during the hypothesized 2002 outbreak, as compared to the 2001 pre-outbreak and the 2003 post-outbreak years. Genetic analysis showed a close relationship between the virus strains, indicating SINV has remained in local annual enzootic circulation since been introduced into the River Dalälven floodplains. We conclude that increased SINV prevalence in vector mosquitoes constitutes a marker most valuable for studying the seven-year outbreak cycle.

## Introduction

Several of the mosquito-borne alphaviruses (Togaviridae) cause disease in humans and they form two groups according to the clinical symptoms in humans. The encephalitis causing alphaviruses occurring only in North and South America, and the arthritogenic alphaviruses with a distribution mainly in tropical and subtropical areas around the World. The arthritogenic alphaviruses (Sindbis virus (SINV), chikungunya virus, Ross River virus, Barmah Forest virus, o´nyong nyong virus, and Mayaro virus), cause endemic disease and occasionally large epidemics [1]. These mosquito-borne diseases present with fever, rash, myalgia, and general peripheral polyarthralgia and/or polyarthritis which is often debilitating and cause long lasting polyarthralgia/polyarthritis in approximately 25% of patients [1].

**Fig 1:**
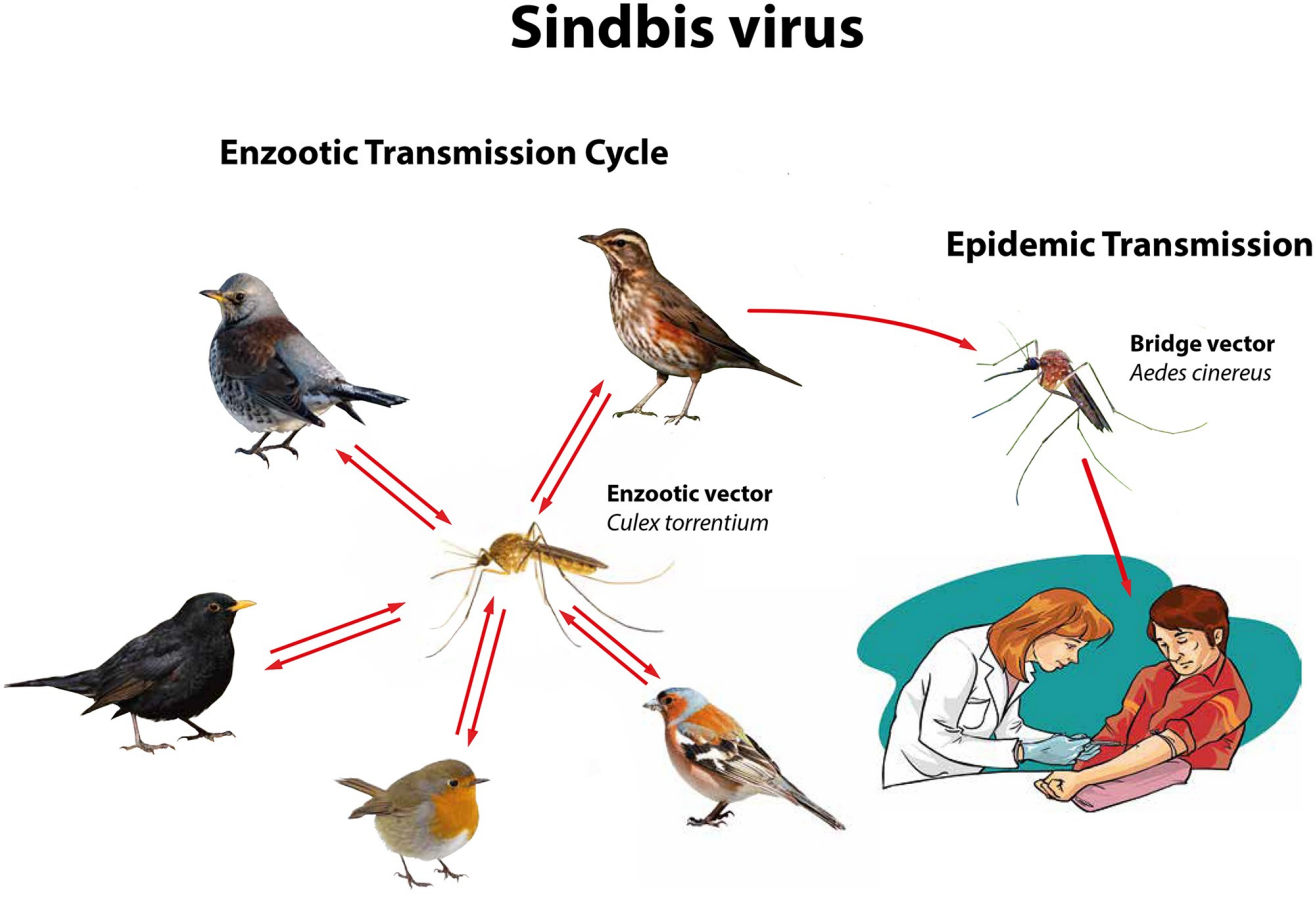
The Sindbis virus enzootic transmission cycle in Sweden involves *Culex torrentium* as main enzootic vector for transmission to thrushes (*Turdus* spp.) as main amplifying hosts. The tangential transmission to humans is mainly by the bridge-vector *Aedes cinereus*, and infected humans are dead-end hosts as their viremia is not high-tittered enough to allow infection of mosquitoes that feed on them.

SINV has a wide geographical distribution in the tropical, sub-tropical and temperate zones of Africa, Asia, Austral-Asia and Europe, forming five genotypes (SINV-I to SINV-V), each restricted to a specific geographical region [2]. Human cases occur in all regions, but outbreaks have only been documented in South Africa and northern Europe [3–7], and are associated with SINV-I [3, 5]. SINV is a zoonosis with birds as amplifying hosts and ornithophilic mosquitoes as vectors, and human are accidentally infected dead-end hosts. A wealth of detailed specific information on the ecology, including enzootic vector species, bridge vector species and amplifying host species, is available for Sweden (Fig 1). The local occurrence of this mosquito-borne and bird-associated zoonosis require SINV-I infection in the main enzootic vector *Culex torrentium* [8–11], and in the main amplifying hosts including redwing *Turdus iliacus*, fieldfare *Turdus pilaris* and other passerines [8, 12–14]. In addition, for tangential spread of SINV-I from viraemic birds to humans, the bridge-vector *Aedes cinereus* needs to be infected and sufficiently abundant [8, 15].

**Fig 2:**
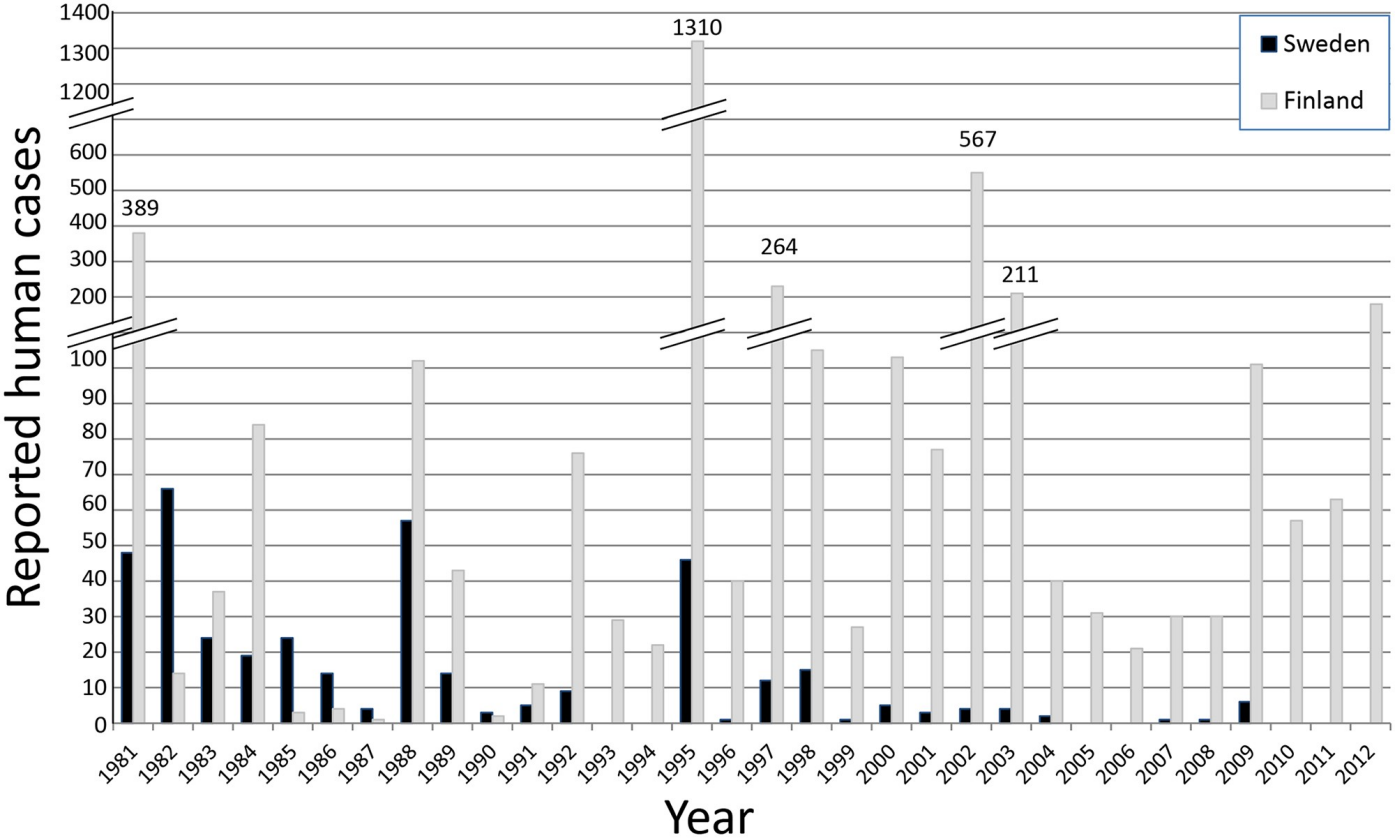
The number of reported and serologically confirmed clinical cases of human Sindbis virus infections 1981 to 2012 in Sweden (Ockelbo disease), and Finland (Pogosta). The considered outbreak years are 1981, 1988, 1995, 2002 and 2009, based on the suggested 7-year cycle between outbreaks.

Human infections with SINV, and other mosquito-borne bird viruses including West Nile virus (WNV), are always preceded by enzootic transmission in bird populations and occurrence of virus-infected vector mosquitoes [16–18]. SINV-I and WNV are ecologically very similar [5], and mosquito surveillance and virus detection in mosquitoes have been suggested as the most reliable and cost-effective method for early WNV outbreak detection [19]. In northern Europe, the first cases of SINV infections were observed 1967 in Sweden and reported as Ockelbo disease, and 1974 in Finland reported as Pogosta [4, 20]. Based on the apparent regular reoccurrence of outbreaks in the 1980s and 1990s, a seven-year outbreak cycle of human SINV infections in northern Europe was suggested [4], and this seven-year cycle is commonly referred to in scientific papers [21–27]. However, the reporting of SINV cases appears to vary in relation to the authorities´ demand for disease diagnosis and registration. In 1981 to 1994 (Fig 2), the number of cases was three times higher in Finland (827 cases) than in Sweden (287 cases). In 1995, clinical SINV infections became notifiable in Finland but not in Sweden, and suddenly the difference between the countries increased dramatically. In the years 1995 to 2012 (Fig 2), the number of cases was 33 times higher in Finland (3350 cases) than in Sweden (101 cases). The regulatory change in 1995 indicates that the authorities´ demand influences the reporting, and that the annual number of reported human SINV cases may neither be adequate for defining a potential seven-year cycle of outbreaks, nor for scrutinising the factors behind the outbreaks. Evidently, there is a need for an objective marker of SINV activity for further studies on the periodicity of outbreaks, and for understanding the causes. From a public health perspective, a defined regularity in the occurrence of outbreaks would be a great advantage and allow the authorities to inform about the risk for mosquito-borne SINV infection with good timing. Such information can motivate people to use protective clothes and repellents to reduce the number of mosquito bites and thereby decrease the risk for infection and disease.

Our aims were to investigate the SINV activity in vector mosquito species, and the abundance of these mosquitoes during transmission season in the years before, during and after an outbreak year, as defined by the suggested seven-year outbreak cycle. The mosquito species in focus are the sibling enzootic vectors *Culex torrentium* and *Culex pipiens* (species not separated, referred to as *Culex torrentium/pipiens*), the potential enzootic vector *Culiseta morsitans*, and the bridge-vector *Aedes cinereus* [8–15]. The mosquito *Aedes rossicus* was included as a potential bridge-vector because it is a close relative of *Aedes cinereus*, and it is active in the floodplains during the transmission season in August and September [28]. An additional aim was to analyse the genetic diversity of SINV strains occurring in the vector mosquito and to understand the introduction and evolution of virus strains over the years (for example by migrating birds).

## Methods

### Study areas

The study was performed in the floodplains of the River Dalälven in central Sweden. These floodplains, containing a mosaic of lakes, wet meadows, marshes, swamps, bogs, deciduous forests, coniferous forests and agricultural areas, harbour at least 30 of the 50 mosquito species recorded for Sweden [11, 28–31]. Serological evidence and occurrence of clinical cases show that the SINV infection is distributed mainly in central Sweden [3], and antibody prevalence in wild bird populations provides a similar pattern [8, 14]. In addition, SINV-I has been isolated from vector mosquitoes sampled in several areas of central Sweden including the River Dalälven floodplains [2, 8, 11].

### Mosquito sampling and identification

**Fig 3:**
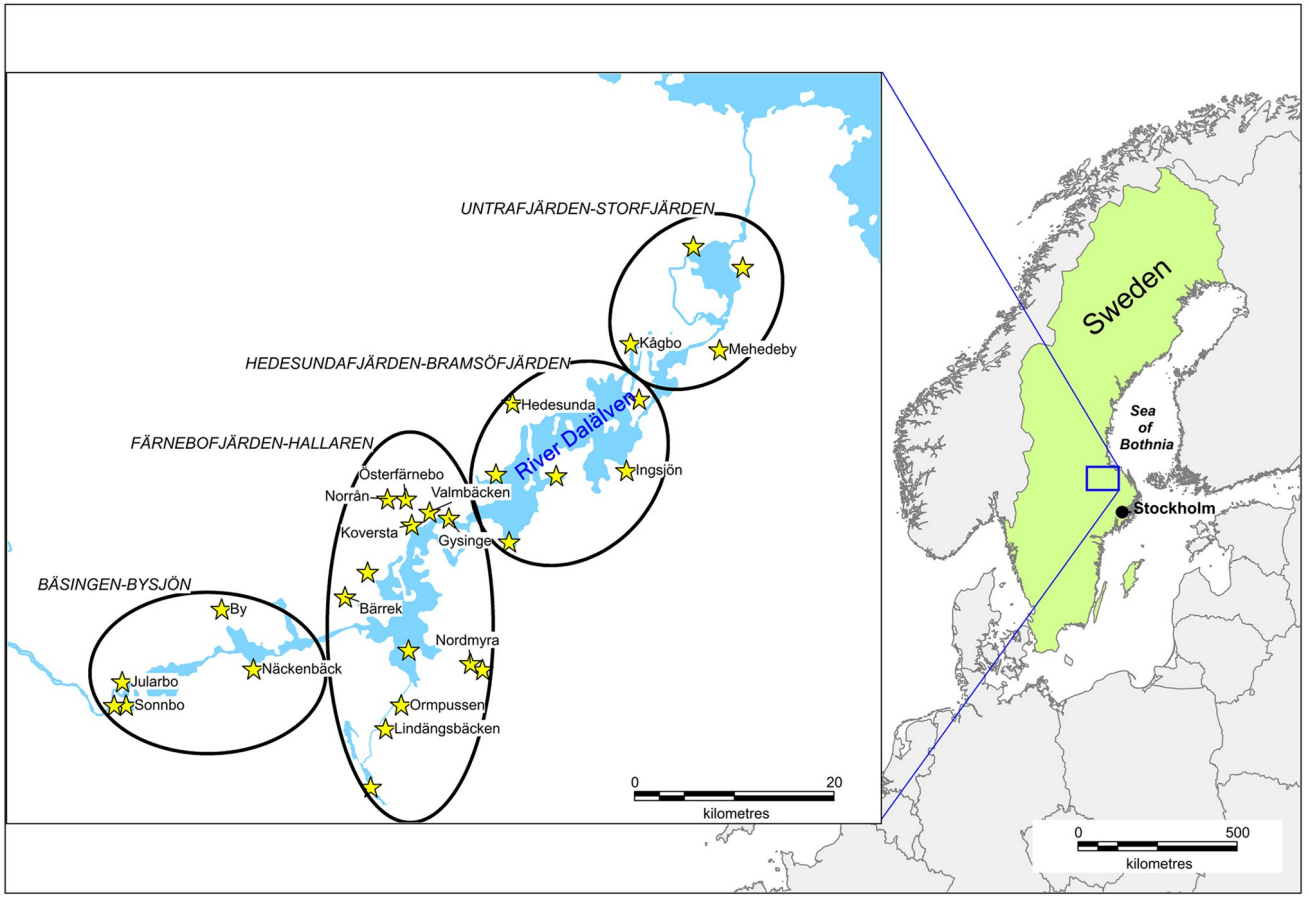
The study sites in the River Dalälven floodplains of central Sweden are located within the endemic area for Ockelbo disease caused by mosquito-borne Sindbis virus. Visualized on the magnified map are the BMC mosquito surveillance (http://www.mygg.se) trap sites with names provided for sites where virus occurred in vector mosquitoes. The ovals show our division of the study sites into four geographic locations named after the major lakes included.

The prevalence of SINV was investigated in blood-seeking female mosquitoes captured within the regional mosquito control operator Biological Mosquito Control (BMC) mosquito surveillance program [http://www.mygg.se]. The mosquito sampling was done around the eight lakes of the River Dalälven floodplains and for our analysis we grouped the mosquito sampling sites into four major geographic location groups named after the lakes; Bäsingen-Bysjön, Färnebofjärden-Hallaren, Hedesundafjärden-Bramsöfjärden, and Untrafjärden-Storfjärden (Fig 3).

The BMC mosquito surveillance program commenced in 2001, which is the year before the 2002 outbreak year according to the suggested seven-year outbreak cycle. For the present study, we focused on vector mosquitoes from 2001 (pre-outbreak year), 2002 (outbreak year) and 2003 (post-outbreak year). The BMC surveillance program utilises Centres for Disease Control and Prevention Miniature Light Traps (CDC-traps, Hausherr´s Machine Works, Toms River, New Jersey, USA) baited with carbon dioxide for sampling blood-seeking female mosquitoes [8], and the sampling is performed biweekly from May (week 19) to September (week 37) providing a total of 10 samples per site and year. One CDC-trap per night and site was used during each week of sampling in 2001 (23 sites, *i*.*e*. 230 trap nights), 2002 (25 sites, *i*.*e*. 250 trap nights) and 2003 (26 sites, *i*.*e*. 260 trap-nights). The large number of study sites required two nights of trapping per sampling week. From each CDC-trap catch, four replicates of 50 mixed species mosquitoes were weighted on a digital scale (Precisa 620C, precision 0.01 g). The average weight was used for calculating the number of mosquitoes per trap and night. All collected mosquitoes were stored at -70°C until identification. Mosquitoes were kept cold on a chill table (custom made) and illuminated by a fiber-optic cold light lamp while examined under a stereoscopic microscope (Olympus SZX7 with 4 to 28 times magnification) for identification based on morphological characters [32]. We identified up to 2000 specimens for each trap and night, as described in Schäfer et al. [28], which corresponds to identification of the full catch for 96% of the 740 trap nights in 2001–2003. Where only a sub-sample was identified we used the proportion of identified species for calculating abundance of each species by trap and night. Identified mosquitoes were pooled by species, year, week, and study site to a maximum of 50 individuals per pool. Only the five established or suspected vector species; *Culex torrentium/pipiens*, *Culiseta morsitans*, *Aedes cinereus*, and *Aedes rossicus* were assayed for SINV.

In addition, SINV prevalence was also investigated in mosquitoes from a previous mosquito diversity study performed over three years in the River Dalälven floodplains [28]. In this study, the mosquito sampling was only performed in six study sites but for each study site we used three CDC-traps that were run for two nights per month (36 trap-nights per month), except in May and September 2000 when three traps were run for three nights (54 trap-nights per month). Sampling was performed May to September in 2000 (216 trap nights), and in 2001 (180 trap nights), and in June to August in 2002 (108 trap nights). Virus screening was performed on all species from 2000, and on only the five potential vector species from 2001 and 2002.

### Virus isolation

Species-identified and pooled mosquitoes, with up to 50 individuals per pool, were transferred into Lysing Matrix D tubes (Peqlab, Erlangen, Germany) containing 1 ml of M199 tissue culture medium, antibiotics, antimycotics solution (ABAM, Invitrogen), and 2% foetal calf serum. The mosquito-fluid mixture was homogenised by two rounds of reciprocation at 5 m/sec for 15 seconds with cooling on ice water between the rounds (FastPrep FP120, BIO 101, Q-BIOgene). The homogenate was pressed through a 45 μm filter (Millipore), before additional 1 mL M199 was pressed through the filter, and 0.2 mL of the filtrate was inoculated into one well of a 6-well plate with confluent Vero B4 cells (LGC Standard, Wesel, Germany). Cells were provided 3 mL M199 with 2% foetal calf serum (Biochrom AG, Berlin, Germany) and incubated at 37°C in 5% CO_2_. Cell cultures were inspected daily, for at least one week, and from wells with observed cytopathogenic effects 0.5 mL of culture supernatant was inoculated into a 25 cm^2^ flask with Vero cells for confirmation of virus-induced cytopathogenic activity.

### Extraction and amplification of viral RNA

A 140 μL aliquot of virus-infected culture supernatant was suspended in AVL buffer for RNA extraction according to the instructions of the manufacturer using the QIAamp Viral RNA Mini Kit (Qiagen, Hilden, Germany). Viral RNA was eluted in a final volume of 40 μL of AVE buffer provided in the kit. For RNA amplification the forward primer SIN-Reg-B: 5’- GCATTCACCTACACCAGTG –3’ (50 μmol), and the reverse primer cSIN-Reg-B: 5’- ATCTAGGAAACTGGTAGTG -3’ (50 μmol), embracing nucleotid positions 8175–10499 of the SINV genome (NCBI Accession number J02363), were used with 5 μL RNA and the Superscript III MasterMix (Invitrogen). We used a one-tube RT-PCR reaction and visualised the product by UV-illumination after ethidium bromide staining [2]. DNA of the correct size of about 2.3 kbp was extracted using the QIAquick PCR Purification Kit according to the manufacturer’s instructions (Qiagen, Hilden, Germany).

### Sequence analysis and phylogenetic relationships

A 4 μL aliquot of the purified amplicon DNA and the BigDye Terminator Cycle Sequencing Ready Reaction Kit (Applied Biosystems) was used for cycle sequencing according to the manufacturer’s instructions. Besides primers SIN-Reg-B and cSIN-Reg-B (see above), primers Sin-Reg-B-nf: 5’- ATGACATCAAGATTAGCACC-3’ (position 8872–8891) and Sin-Reg-B-nr: 5’- TGATGCGACGGCTAAG –3’ (position 9734–9749) were used in sequencing reactions to reach full redundancy. The reaction was purified using Centri-Sep columns as recommended (Princeton Separations Inc, Adelphia, USA) and analysed by an ABI PRISM 310 Genetic Analyser (Applied Biosystems).

Sequences were aligned using GramAlign v3.0 [33]. A Maximum clade credibility (MCC) tree with dated tips and internal nodes was inferred using a MCMC Bayesian approach under the GTR model with gamma-distributed rate variation (Γ) and a proportion of invariable sites (I) using a relaxed (uncorrelated lognormal) molecular clock [34] in BEAST version 1.8.4 [35]. Four independent MCMC runs of four chains each were run for 10,000,000 states. The First 1,000,000 were used as burning and the MCC was establish from the remaining states. A median joining network [36] of the sequences was constructed and edited using a 2,190-character set in SPLITSTREE v4.12.3 [37]

### Statistical analysis

To remove the differences in sampling, we only used data from the 2001–2003 biweekly sampling for the statistical analysis. We calculated the Maximum Likelihood Estimate Infection Rate (MLE-IR) for each of the species individually, as well as for the group of enzootic vectors (*Culex torrentium/pipiens*, *Culiseta morsitans*) and for the group of bridge-vectors (*Aedes cinerus*, *Aedes rossicus*), respectively. MLE-IR is presented as estimated number of mosquitos with positive SINV detection out of 1000 mosquitos, i.e. Maximum likelihood detection rate * 1000. We used the Excel-macro available at CDC’s Mosquito Surveillance Software [https://www.cdc.gov/westnile/resourcepages/mosqSurvSoft.html]. This Excel-macro was also used to analyse if MLE-IR differed *between* two species or two groups of species (enzootic and bridge-vectors, respectively).

To study temporal (year, *t*) and spatial (geographic location group, *i*; Fig 3) variation in infection rate between species or groups of species, *x*, we used MLE-IR_*x*,*t*,*i*_ for a species *x* in year *t* and geographic location group *i*MLE-IR_*x*,*t*,*i*_ was used as response variable in two-way ANOVA with year and geographic location group as class variables (hence, total number of replicates is twelve). To increase the statistical power an insignificant variable was removed if P > 0.1. A significant difference indicates that the probability to detect SIN virus is not equal between years and geographic location groups.

Finally, to study associations between infection rates and mosquito abundance among years and geographic location groups, we did Pearson correlations analysis between MLE-IR_*x*,*t*,*i*_ and the estimated number of mosquitoes per trap and night for each year and geographic location group for all species or species groups separately. A significant correlation indicates that the probability to detect SINV in a species is associated with the mosquito abundance.

## Results

### Virus in mosquitoes by sampling strategy

Twenty SINV strains were isolated from the five species screened from the 2001–2003 biweekly sampling in the floodplains of the River Dalälven (Tables 1 & 2).

**Table 1:** Sampling characteristics of the 22 Sindbis virus strains isolated from mosquitoes collected 2000 to 2003 in the River Dalälven floodplains, central Sweden. The mosquitoes were collected in two separate studies; monthly sampling at six study sites in 2000–2002 for a mosquito diversity study [28], and biweekly sampling in 23–26 study sites in 2001–2003 as part of the BMC mosquito surveillance program (http://www.mygg.se).

Study	Species	Pool size	Study site	Date sampled	Strain	Accession number
**Monthly**	*Aedes cinereus*	43	Valmbäcken	Sept 19, 2000	B429	KM880164
	*Aedes rossicus*	50	Valmbäcken	Aug 16, 2002	E239	KM880171
**Biweekly**	*Culex torrentium/pipiens*	28	Several sites^a^	Jul 31 & Aug 2, 2001	D009	KM880165
	*Culex torrentium/pipiens*	18	Several sites^b^	Aug 01, 2002	E099	KM880168
	*Culex torrentium/pipiens*	6	Several sites^c^	Aug 13, 2002	E102	KM880169
	*Culex torrentium/pipiens*	12	Several sites^d^	Aug 13, 2002	E917	KM880180
	*Culiseta morsitans*	2	Norrån	Aug 01, 2002	E066	KM880167
	*Culiseta morsitans*	5	Several sites^e^	Aug 27, 2002	E107	KM880170
	*Aedes cinereus*	50	Ingsjön	Aug 02, 2002	E424	KM880172
	*Aedes cinereus*	50	Norrån	Aug 13, 2002	E029	KM880166
	*Aedes cinereus*	50	Nordmyra	Aug 13, 2002	E426	KM880173
	*Aedes cinereus*	50	Ormpussen	Aug 27, 2002	E499	KM880174
	*Aedes cinereus*	50	By	Aug 27, 2002	E522	KM880175
	*Aedes cinereus*	50	Mehedeby	Aug 27, 2002	E533	KM880176
	*Aedes cinereus*	50	Ormpussen	Sept 10, 2002	E585	KM880177
	*Aedes rossicus*	50	Ormpussen	Sept 10, 2002	E594	KM880178
	*Aedes rossicus*	50	Lindängsbäcken	Sept 10, 2002	E597	KM880179
	*Aedes rossicus*	46	Several sites^f^	Sept 10, 2002	E945	KM880181
	*Aedes cinereus*	50	Kågbo	Jul 30, 2003	F231	KM880182
	*Aedes cinereus*	50	Ormpussen	Aug 11, 2003	F251	KM880183
	*Aedes cinereus*	9	Valmbäcken	Aug 27, 2003	F281	KM880184
	*Aedes cinereus*	19	Mehedeby	Aug 27, 2003	F384	KM880185

^a^ These 28 mosquitoes were from Nordmyra (1), By (1), Bärrek (2), Österfärnebo (4), Norrån (18), Gysinge (1) and Hedesunda (1) in the Färnebofjärden-Hallaren area.

^b^ These 18 mosquitoes were from Österfärnebo (2), Koversta (3), Norrån (11), and Gysinge (2) in the Färnebofjärden-Hallaren area.

^c^ These 6 mosquitoes were from Koversta (1), Norrån (3), and Österfärnebo (2) in the Färnebofjärden-Hallaren area.

^d^ These 12 mosquitoes were from Näckenbäck (1), Sonnbo (2), By (3), and Jularbo (6) in the Bäsingen-Bysjön area.

^e^ These 5 mosquitoes were from Norrån (2), Koversta (2), and Österfärnebo (1) in the Färnebofjärden-Hallaren area.

^f^ These 46 mosquitoes were from Sonnbo (3), and Näckenbäck (43) in the Bäsingen-Bysjön area.

**Table 2:** Sindbis virus infection rates in biweekly samples of vector mosquito species collected in the River Dalälven floodplains, central Sweden, during 2001 until 2003.

Species or group	Time period^a^	2001	2002	2003	All years
*Culex torrentium/pipiens*	Early season	0/130 (0.0)^b^	0/44 (0.0)	0/101 (0.0)	0/277 (0)
	Late season	1/81 (13.6)	3/247 (11.9)	0/48 (0.0)	4/374 (10.9)
	**Full season**	**1/211 (4.9)**	**3/291 (10.0)**	**0/149 (0)**	**4/651 (6.2)**
*Culiseta morsitans*	Early season	0/12 (0.0)	0/18 (0.0)	0/3 (0.0)	0/35 (0.0)
	Late season	0/241 (0.0)	2/202 (9.8)	0/91 (0.0)	2/533 (3.7)
	**Full season**	**0/253 (0.0)**	**2/220 (9.0)**	**0/94 (0.0)**	**2/568 (3.5)**
Total enzootic vectors	Early season	0/142 (0.0)	0/62 (0.0)	0/104 (0.0)	0/312 (0.0)
	Late season	1/322 (3.2)	5/449 (11.1)	0/139 (0.0)	6/907 (6.7)
	**Full season**	**1/464 (2.2)**	**5/511 (9.7)**	**0/243 (0)**	**6/1219 (4.9)**
*Aedes cinereus*	Early season	0/9262 (0.0)	0/1754 (0.0)	0/6262 (0.0)	0/17,278 (0.0)
	Late season	0/3407 (0.0)	7/9966 (0.73)	4/6369 (0.63)	11/19,742 (0.57)
	**Full season**	**0/12,669 (0.0)**	**7/11,720 (0.62)**	**4/12,631 (0.32)**	**11/37,020 (0.30)**
*Aedes rossicus*	Early season	0/4235 (0.0)	0/2258 (0.0)	0/480 (0.0)	0/6973 (0.0)
	Late season	0/2765 (0.0)	3/8164 (0.37)	0/543 (0.0)	3/11,472 (0.26)
	**Full season**	**0/7000 (0.0)**	**3/10,422 (0.29)**	**0/1023 (0.0)**	**3/18,445 (0.16)**
Total bridge-vectors	Early season	0/13,497 (0.0)	0/4012 (0.0)	0/6742 (0.0)	0/24,251 (0.0)
	Late season	0/6172 (0.0)	10/18,130 (0.57)	4/6912 (0.58)	14/31,214 (0.46)
	**Full season**	**0/19,669 (0.0)**	**10/22,142 (0.46)**	**4/13,654 (0.29)**	**14/55,465 (0.26)**

^a^ Early season is week 19–28, late season is week 29–37, and full season is 19–37.

^b^ Number of virus isolates / number of mosquitoes assayed (Maximum Likelihood Estimate Infection Rate/1000 mosquitoes).

One strain was obtained in 2001, 15 in 2002, and 4 in 2003, and all the isolated strains originate from mosquitoes collected between July 30 and September 10 (Table 1). In contrast, the 2000–2002 monthly sampling only resulted in isolation of two SINV-1 strains, also collected in late summer (Tables 1 & 3).

**Table 3:** Sindbis virus infection rates in monthly samples of vector mosquito species collected in the River Dalälven floodplains, central Sweden, during 2000 until 2002.

Species or group	Time period^a^	2000	2001	2002	All years
*Culex torrentium/pipiens*	Early season	0/22 (0.0)^b^	0/5 (0.0)9	0/21 (0.0)	0/102 (0.0)
	Late season	0/4 (0.0)	0/18 (0.0)	0/52 (0.0)	0/74 (0.0)
	**Full season**	**0/26 (0.0)**	**0/77 (0.0)**	**0/73 (0.0)**	**0/176 (0.0)**
*Culiseta morsitans*	Early season	0/37 (0.0)	0/9 (0.0)	0/37 (0.0)	0/83 (0.0)
	Late season	0/125 (0.0)	0/108 (0.0)	0/64 (0.0)	0/297 (0.0)
	**Full season**	**0/162 (0.0)**	**0/117 (0.0)**	**0/101 (0.0)**	**0/380 (0.0)**
Total enzootic vectors	Early season	0/59 (0.0)	0/68 (0.0)	0/58 (0.0)	0/185 (0.0)
	Late season	0/129 (0.0)	0/126 (0.0)	0/116 (0.0)	0/371 (0.0)
	**Full season**	**0/188 (0.0)**	**0/194 (0.0)**	**0/174 (0.0)**	**0/556 (0.0)**
*Aedes cinereus*	Early season	0/2568 (0.0)	0/6870 (0.0)	0/2567 (0.0)	0/12,005 (0.0)
	Late season	1/11,184 (0.09)	0/1982 (0.0)	0/5112 (0.0)	1/18,278 (0.05)
	**Full season**	**1/13,752 (0.07)**	**0/8852 (0.0)**	**0/7679 (0.0)**	**1/30,283 (0.03)**
*Aedes rossicus*	Early season	0/3570 (0.0)	0/2295 (0.0)	0/1177 (0.0)	0/7042 (0.0)
	Late season	0/16,985 (0.0)	0/2231 (0.0)	1/4907 (0.20)	1/24,123 (0.04)
	**Full season**	**0/20,555 (0.0)**	**0/4526 (0.0)**	**1/6084 (0.16)**	**1/31,165 (0.03)**
Total bridge-vectors	Early season	0/6138 (0.0)	0/9353 (0.0)	0/3744 (0.0)	0/19,235 (0.0)
	Late season	1/28,169 (0.04)	0/4213 (0.0)	1/10,019 (0.10)	2/42,401 (0.05)
	**Full season**	**1/34,307 (0.03)**	**0/13,566 (0.0)**	**1/19,372 (0.05)**	**2/67,245 (0.03)**

^a^ Early season is week 21–28 in 2000, week 20–28 in 2001, and week 24–28 in 2002. Late season is week 33–38 in 2000, week 32–36 in 2001, and week 33 in 2002. Full season is week 21–38 in 2000, week 20–36 in 2001, and week 24–33 in 2002.

^b^ Number of virus isolates/number of mosquitoes assayed (Maximum Likelihood Estimate Infection Rate/1000 mosquitoes).

We obtained more SINV strains from the biweekly than from the monthly samples in both 2001 (biweekly 1 isolate, monthly 0 isolates) and 2002 (biweekly 15 isolates, monthly 1 isolate). Out of the 22 species screened in 2000, no additional vector species were identified (S1 Supplementary information). We consider the period from middle of July until the end of mosquito sampling in September as the main SINV activity season, because all virus isolates were obtained from mosquitoes collected between July 30 (week 33) and September 10 (week 37). Therefore, we show the number of mosquitoes tested for virus, the number of virus strains isolated, and the infection rate per 1,000 mosquitoes for early season (week 19–28), late season (week 29–38) and full season (week 19–38, see below and Tables 2 and 3).

### SINV infection by mosquito species, vector species group and time

Mosquito infection rate varied between the two vector species groups, and between the five vector species (Table 2). SINV was more prevalent in the enzootic vectors (MLE-IR = 4.9/1000), than in the bridge-vectors (MLE-IR = 0.26/1000) (99% CI = [1.5, 12.1]). The main enzootic vectors *Culex torrentium/pipiens*, with MLE-IR of 4.9/1000 in 2001 and MLE-IR = 10.0/1000 in 2002, showed the highest infection probability. Among the bridge-vectors, SINV was more prevalent in *Aedes cinereus* (MLE-IR = 0.30/1000) than in *Aedes rossicus* (MLE-IR = 0.16/1000) but the difference was not significant (Diff = 0.14, 95% CI = [-0.17, 0.4]).

The total number of SINV strains in vector mosquitoes was highest in 2002 (Table 2), and MLE-IR_t,*i*_ showed a near-significant difference between years (ANOVA: F_2,9_ = 5.5, P = 0.028, N = 12). The infection rate (MLE-IR_*x*,*t*,*i*_) in the bridge-vectors alone (*Aedes* ssp.) was higher in 2002 (ANOVA F_2,9_ = 7.3, P = 0.01, N = 12), than in the other years. Geographic location within the River Dalälven floodplains had no significant effect on the infection rates (MLE-IR_*x*,*t*,*i*_) for neither the vector species tested nor for the vector species groups tested (ANOVA: F_3,6_ < 1.1, P > 0.4 for both), and was omitted from the final ANOVA.

MLE-IR_*t*,*i*_ (all species as one group) was neither correlated to the number of vector mosquitoes tested for virus, nor to the abundance of the vector mosquitoes in each geographic location group and year (rp < 0.35, P > 0.25 for both). Interestingly, the annual abundance of *Culex torrentium/pipiens* showed marginal significant positive correlation with MLE-IR_*x*,*t*,*i*_ in *Aedes rossicus* (rp = 0.55, P = 0.063, N = 12).

### Mosquito abundance and infection

The biweekly abundance of *Culex torrentium/pipiens* varied between 0.1 and 2.0 per trap-night, with little variation among or within years, except for a sharp increase to 8.9 per trap-night in week 31, 2002. The MLE-IR was 4.9/1000 in 2001, and 10/1000 in 2002, but no virus was detected in the 149 *Culex torrentium/pipens* collected in 2003 (Table 2).

**Fig 4:**
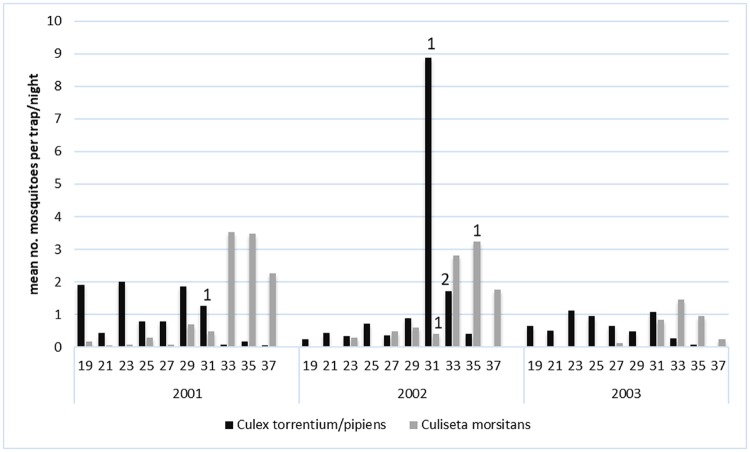
Temporal occurrence of the enzootic vectors *Culex torrentium/pipiens* and *Culiseta morsitans*, and of Sindbis virus isolated from these mosquitoes, during the summer seasons of 2001 to 2003 in the floodplains of River Dalälven, central Sweden. The bars refer to biweekly mosquito abundance and the numbers on top of bars refer to virus isolations.

The biweekly abundance of *Culiseta morsitans* varied between 0.1 and 3.5 per trap-night (Fig 4), with an annual peak of activity in late August (week 33–35). SINV isolates from *Culiseta morsitans* were only obtained in August 2002 (Table 2, Fig 4).

**Fig 5:**
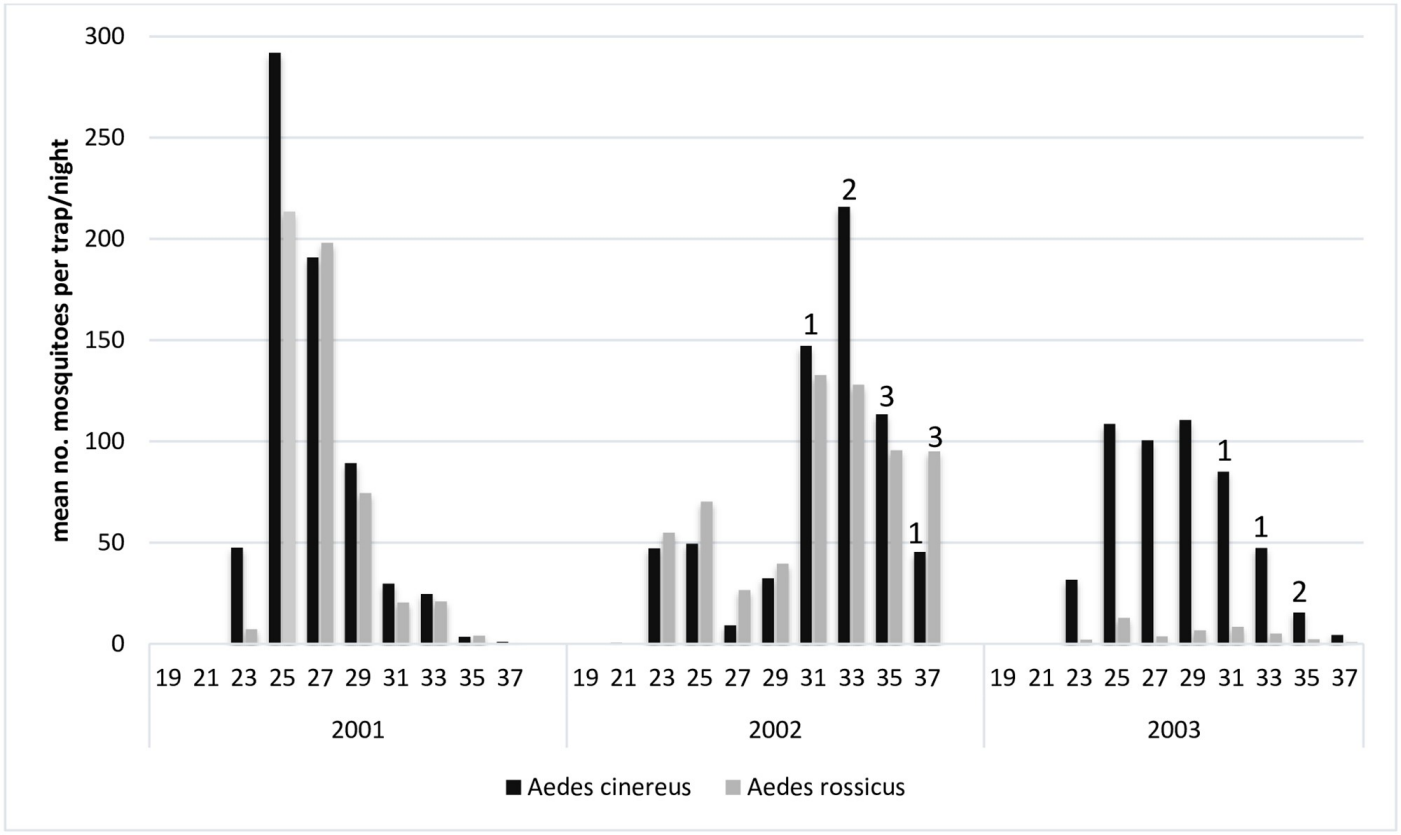
Temporal occurrence of the bridge-vectors *Aedes cinereus* and *Aedes rossicus*, and of Sindbis virus in the mosquitoes, during the summer seasons of 2001 to 2003 in the floodplains of River Dalälven, central Sweden. The bars refer to biweekly mosquito abundance and the numbers on top of bars refer to virus isolations.

The biweekly abundance of *Aedes cinereus* varied within season, and the peak abundance was rather similar between the years studied but with variable timing (Fig 5). In 2001, a peak abundance of 290 per trap-night was reached already in week 25. In the next two years, peak abundance occurred later, with 230 per trap-night in week 33 in 2002, and 110 per trap-night at week 29 in 2003. About 12,000 *Aedes cinereus* were collected and assayed each year (Table 2), resulting in no detectable virus in the pre-outbreak year 2001, an MLE-IR of 0.60/1000 in the outbreak year 2002, and 0.32/1000 in the post-outbreak year 2003. The activity pattern for *Aedes rossicus* and *Aedes cinereus* was very similar, with the exception for 2003 when *Aedes rossicus* occurred in very low abundance (Fig 5).

Virus isolates from *Aedes rossicus* were obtained in August and September 2002, and these were the first virus isolates from the species (Table 1, Fig 5).

### Virus strain characterisation and phylogeny

For the 22 Swedish SINV strains we determined 2190 nucleotides (nt) or 730 amino acids (aa) covering the coding region for the carboxy-terminal of the capsid (186 nt, 62 aa), the entire E3 (192 nt, 64 aa), E2 (1269 nt, 423 aa), and 6K (165 nt, 55 aa) proteins, as well as the n-terminal of the E1 protein (378 nt, 126 aa). Compared to the corresponding nt of the prototype SIN virus EgAr339, there were differences at 189 positions (8.6%), and at 125 of these positions all 22 isolates had identical nucleotides differing from the prototype EgAR339. Out of the 64 additional sites with nucleotide changes, 34 were a single nucleotide polymorphism neither shared by any of the other strains nor the reference strain, with further seven positions having mutations shared by only two strains. The observed nucleotide mutations were equally distributed over the genomic range investigated. Corresponding NCBI Accession numbers (KM880164-KM880165) are provided (Table 1).

We deduced 730 aa, of the total 1245 aa in the entire structural proteins and observed differences from prototype SINV at 33 aa positions (4.5%). All the Swedish strains had identical aa changes at 18 sites, with unique changes at 12 sites, leaving only three positions where two or more strains showed different aa (Table 4).

**Table 4:** Deduced amino acids in the 22 SINV strains from mosquitoes collected 2000–2003 in the River Dalälven floodplains, central Sweden, showing the similarities and differences to the prototype SINV strain EgAR339 (NCBI Accession number NC_001547.1) within the analysed part of the structural genes. Amino acid positions in bold are shared among all 22 Swedish strains but differ from the SINV type strain.

	Amino acid positions with exchanges in genes																																
	C	E3			E2																					6K				E1			
strain^a^	249	7	20	46	3	5	29	55	61	69	70	75	89	116	126	134	172	212	247	312	314	375	386	408	418	1	20	29	41	43	60	113	116
SINV	S	T	D	A	I	G	V	Q	A	L	K	V	G	V	L	F	R	S	D	V	K	T	V	I	V	E	V	F	P	N	I	V	S
B429			N		T	D	I	K	T	F	E			A	M		G	M	A	I		A	A					V	L		V	D	T
D009			N		T	D	I	K	T	F	E			A	M		G	T	A	I		A	A					_**°**_			V	D	T
E029			N		T	D	I	K	T	F	E			A	M		G	T	A	I		A	A					V			V	D	T
E066			N		T	D	I	K	T	F	E			A	M		G	T	A	I		A	A	V				V			V	D	T
E099			N		T	D	I	K	T	F	E			A	M		G	T	A	I		A	A					V			V	D	T
E102			N		T	D	I	K	T	F	E			A	M		G	T	A	I		A	A			G	_**°**_	V			V	D	T
E107			N		T	D	I	K	T	F	E			A	M		G	T	A	I		A	A					V			V	D	T
E239	G		N		T	D	I	K	T	F	E			A	M		G	T	A	I		A	A					V			V	D	T
E424			N		T	D	I	K	T	F	E			A	M		G	T	A	I		A	A					V		S	V	D	T
E426			N		T	D	I	K	T	F	E			A	M		G	T	A	I		A	A					V		S	V	D	T
E499			N		T	D	I	K	T	F	E			A	M		G	T	A	I		A	A					V			V	D	T
E522			N		T	D	I	K	T	F	E			A	M		G	T	A	I		A	A					V			V	D	T
E533			N		T	D	I	K	T	F	E			A	M		G	T	A	I		A	A					V			V	D	T
E585		M	N		T	D	I	K	T	F	E			A	M	I	G	T	A	I		A	A					I			V	D	T
E594			N		T	D	I	K	T	F	E			A	M		G	T	A	I		A	A					V			V	D	T
E597			N		T	D	I	K	T	F	E			A	M		G	T	A	I		A	A					V			V	D	T
E917			N		T	D	I	K	T	F	E			A	M		G	T	A	I		A	A		I			V			V	D	T
E945			N		T	D	I	K	T	F	E			A	M		G	T	A	I		A	A					V			V	D	T
F231			N		T	D	I	K	T	F	E			A	M		G	T	A	I		A	A					V			V	D	T
F251			N		T	D	I	K	T	F	E	I	R	A	M		G	A	A	I		A	A					V			V	D	T
F281			N	D	T	D	I	K	T	F	E			A	M		G	T	A	I		A	A					V			V	D	T
F384			N		T	D	I	K	T	F	E			A	M		G	T	A	I	R	A	A				A				V	D	T

^a^ = for strain numbering see Table 1, SINV is the type strain (NCBI Accession number NC_001547.1)

Maximum likelihood analysis showed that all the 22 SINV isolates from central Sweden are of the SINV genotype I (S2 Supplementary information).

**Fig 6:**
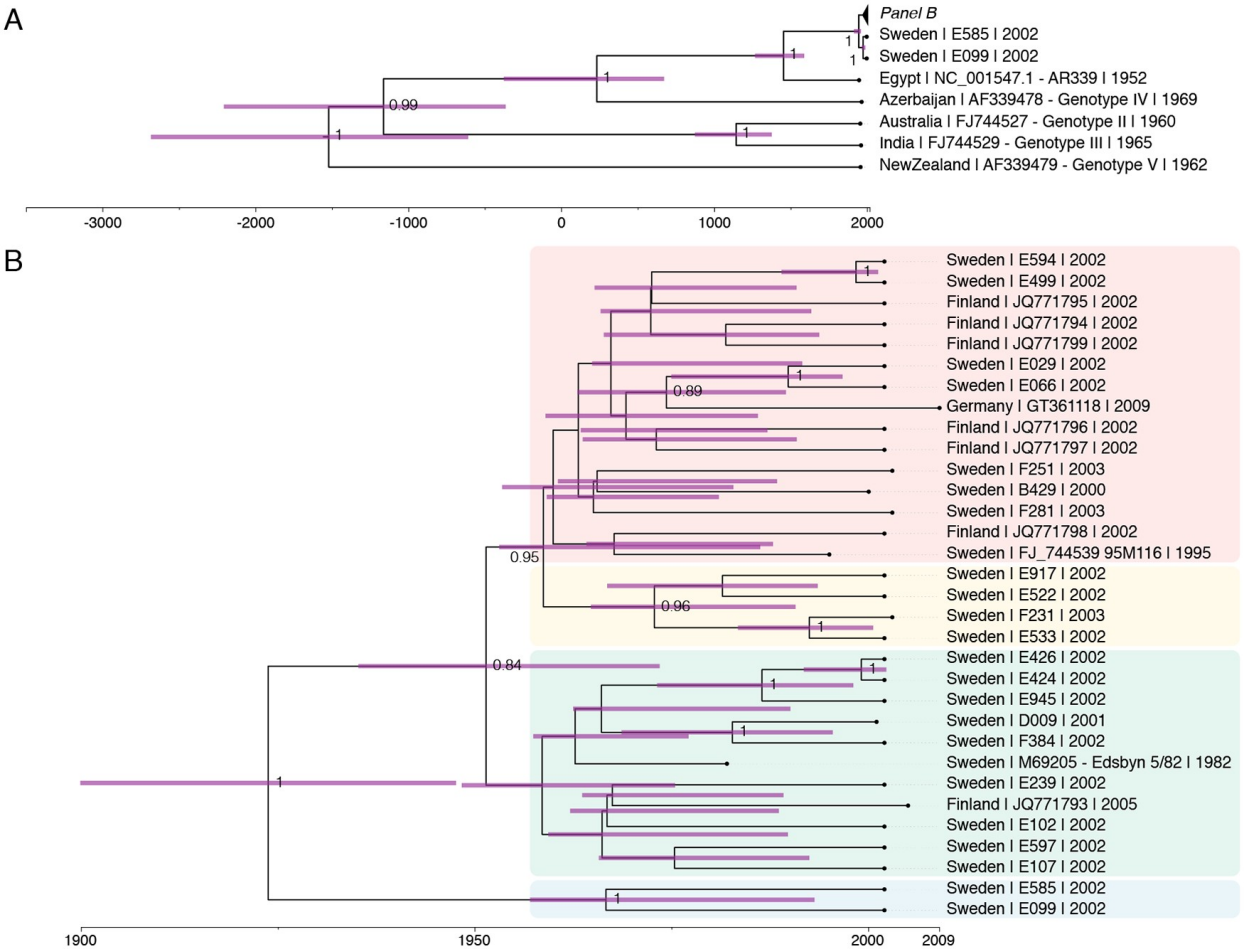
Maximum clade creditability tree obtained from four independent Markow Chain Monte Carlo runs of 22 partial Sindbis virus sequences of strains from Sweden, Finland, Germany, Egypt, Azerbaijan, Australia, India and New Zealand: A) the major branching of five Sindbis genotypes with all Swedish strains in genotype I (SINV-I), and with a time line showing that the most basic branching occurred approximately 2000 years ago. B) Detail of panel B showing the branching patterns among SINV-I strains from Sweden, Finland and Germany, and with a timeline showing that the most basic branching occurred already in the first half of the previous century. The black lines show the branching pattern and the horizontal purple lines show standard deviations of time estimates for each nod.

The maximum clade credibility (MCC) tree obtained from four independent Markov-Chain Monte Carlo runs inferred for the 22 SINV isolates of this study suggests a history of about 2000 years of SINV evolution, while the evolution of SINV detected in Sweden is rather more recent with the majority of strains detected emerging in the 1970s (Fig 6A). High posterior probability values subdivide the Scandinavian section of the MCC tree into 4 clades presenting with deep branch lengths (Fig 6B). This structure indicates that 4 subclades of SINV appear to have been introduced into Scandinavia and have been evolving in local transmission cycles since. This is corroborated by Finnish sequences reported from an outbreak in 2002 which group into clade 1 and 3.

**Fig 7:**
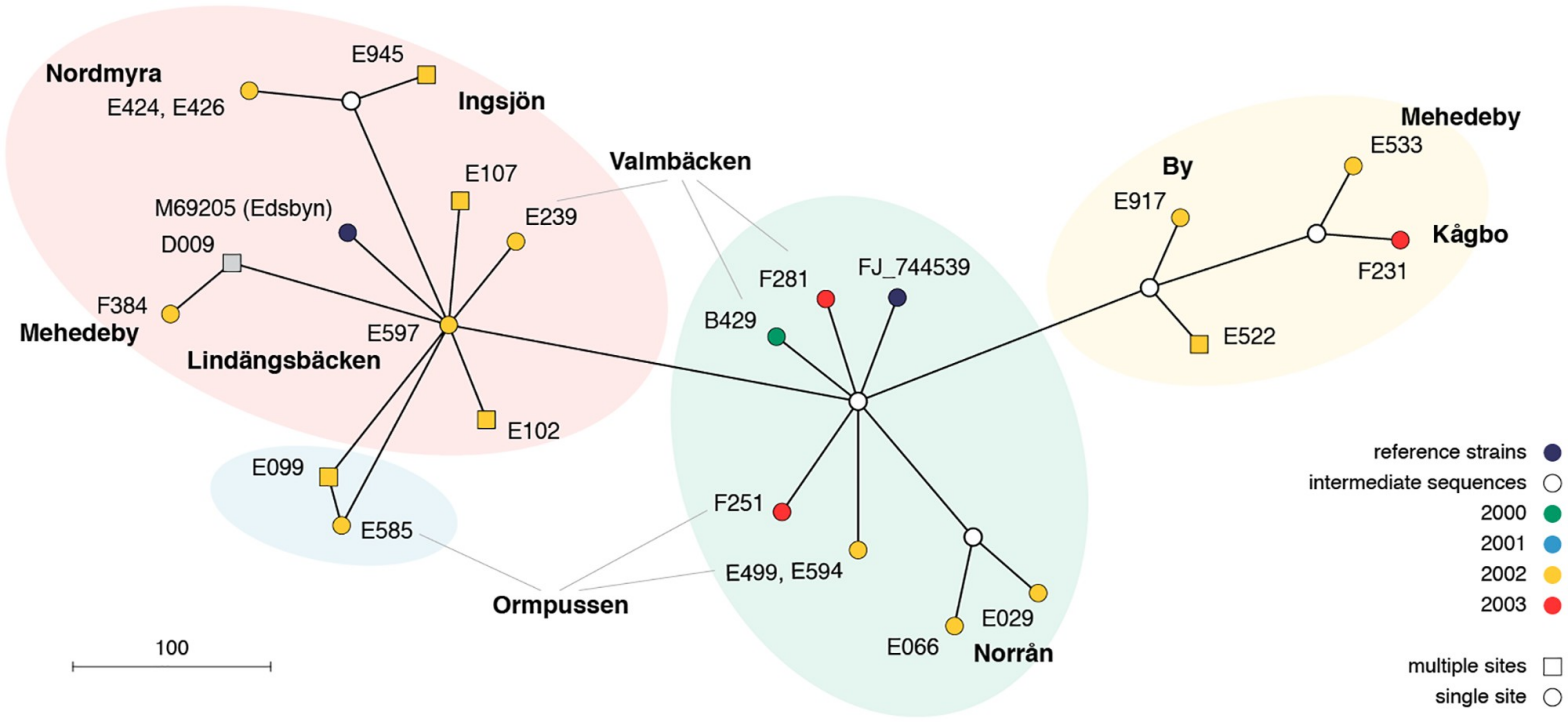
Median joining network analysis of partial sequences of Sindbis virus strains from the River Dalälven floodplains in central Sweden.

The network analysis and the sequence of SINV strain appearance over the years suggests that there is a great diversity of strains present in the mosquito population of the River Dalälven floodplains in central Sweden, with many SINV strains occurring at several sites and several SINV strains occurring at one site (Fig 7). In essence the four clades do not show any distinct geographical distribution indicating diverse SINV evolution in a successful transmission among birds by the local mosquito population.

## Discussion

Our results suggest that increased SINV-I prevalence in the bridge-vector *Aedes cinereus* and in the enzootic vectors *Culex torrentium/pipiens* constitutes objective markers for outbreaks of SINV polyarthritis and rash in northern Europe. We observed low SINV-I prevalence in vector mosquitoes in the pre-outbreak in 2001 (1 isolate, from enzootic vectors), a sharp increase during the expected outbreak in 2002 (15 isolates, 5 from enzootic vectors and 10 from bridge-vectors), and a decline in the post-outbreak year 2003 (4 isolates, all from bridge-vectors). Although only one triad of pre-outbreak, outbreak and post-outbreak years was studied, we observed significantly increased SINV prevalence in the bridge-vectors in the 2002 outbreak year. The additional results from 2009, the next expected outbreak year, again showed increased virus activity in vector mosquitoes (16 isolates from enzootic vectors) [26]. The documented higher SINV prevalence in vector mosquitoes from the River Dalälven floodplains during the outbreak years 2002 and 2009 than during inter-epidemic years provides the first objective markers for SINV polyarthritis outbreaks in northern Europe.

The observed increased SINV-I activity in vector mosquitoes during 2002 and 2009 coincides with the hypothesised seven-year reoccurrence of outbreaks, although the picture is less clear when comparing the annual number of reported disease cases in Sweden. Interestingly, the distinctly higher SINV prevalence in enzootic vector mosquitoes during the 2009 than the 2002 outbreak year, is mirrored in a sharply increased SINV antibody prevalence in the main amplifying host population. In 2009, the *Culex torrentium/pipiens* infection rate was very high at 21.0/1000 [11], and so was the *Turdus* antibody prevalence at 65,4% (n = 127) [38]. In 2002, the *Culex torrentium/pipiens* infection rate was high at 10.0/1000 [present study], and the amplifying host antibody prevalence was much lower with only 1.5% (n = 68) [38]. Evidently, a distinct SINV prevalence increase in the enzootic vectors is connected to a likewise distinct increase in the SINV antibody prevalence in the main amplifying host species, indicating that higher SINV prevalence in enzootic vectors provides a scaled outbreak risk signal.

In Sweden, clinical SINV cases have distinct temporal occurrence in July until October, with a peak in second half of August [3], and mainly floodwater mosquitoes of a few species are active in August and September when the majority of Ockelbo disease cases occur [3, 28]. We collected vector mosquitoes from May to September in 2000–2003 for virus assay and found that SINV were detectable in mosquitoes in the time period of July 30 to September 10. Similarly, in our previous study of mosquito-borne viruses in central Sweden, SINV was only detected in the time period of July 16^th^ to August 30^th^ although larger number of mosquitoes were tested from the early part of summer [8]. Thus, the activity of SINV-I also has a distinct temporal pattern, with the majority of virus isolates obtained from vector mosquitoes in August, a few in September, but not a single isolate in May, June and the first weeks of July. These results show that virus detection in vector mosquitoes, if collected during middle of July until September, will provide reliable information on virus activity without the costs and efforts of full season sampling. More focused sampling will also reduce the number of mosquitoes for identification, and the number of mosquito pools to assay for virus. Thus, focused vector mosquito sampling and virus testing can reduce the costs for collecting the objective information suitable for testing the hypothesis of a seven-year cycle of SINV polyarthritis and rash outbreaks in northern Europe. It is also providing a platform for further scrutinising the mechanisms behind the suggested regular cyclic reoccurrence of outbreaks.

The floodwater mosquito *Aedes cinereus* is the main bridge-vector infecting humans with SINV. This conclusion is based on the proven vector competence for SINV as shown in infection and transmission experiments, and the temporal co-occurrence of virus in this mosquito and of human cases [8, 15, 17]. Therefore, almost every human case of Ockelbo disease is likely the result of a SINV-infected *Aedes cinereus* taking a human blood-meal during August or September, when mosquitoes are few but infective. Large outbreaks of SINV-induced polyarthritis and rash probably require massively increased enzootic SINV-I activity in both vectors and hosts, and that the main bridge-vector *Aedes cinereus* is infective and sufficiently abundant in August to September. The SINV infection rate of 0.62/1000 *Aedes cinereus* in the present study, and 0.09/1000 in the previous study [8], apparently is sufficient for making it an efficient bridge-vector. Thus, detection of SINV in the bridge vector *Aedes cinereus* gives a reliable representation of the risk for SINV infections in humans and thereby provide information for outbreak risk evaluation.

Seasonality in virus activity, with maximum prevalence in vectors at the end of mosquito season, explains the discrepancy between the abundance of strains obtained from vector mosquitoes in our two parallel studies in the same geographic area during 2002. The monthly sampling in 2002 provided only one SINV isolate from the 10,135 vector mosquitoes (infection rate 0.10/1000) collected in 12 trap-nights late in the season (one sampling occasion August 14^th^ and 16^th^). In contrast, the biweekly sampling provided ten isolates from the 22,142 vector mosquitoes (infection rate 0.45/1000) collected in 115 trap-nights late in the season (five sampling occasions July 16^th^ to September 11^th^). Thus, vector mosquito sampling and testing for SINV surveillance in northern Europe should focus on obtaining sufficiently large samples in the late season, since virus prevalence in vector mosquitoes is at its maximum in August and September. However, if future studies aim for an early outbreak risk signal the focus should be on sampling the vector mosquitoes in July. In each of the three years studied we first detected SINV activity in week 31 (1 isolate 2001, 3 isolates 2002, 1 isolate 2003) which includes last days of July. This is just about the same time as the first human cases show up, indicating that larger numbers of vector mosquitoes need to be sampled earlier in July if virus detection is to be used as an early signal. Further development of a strategy for early detection should therefore sample large numbers of vector mosquitoes in the whole of July when the virus population is building up. This could be achieved by weekly sampling with multiple traps in a few suitable sites rather than the present biweekly sampling with single traps in multiple sites over a very large geographic area.

The potential bridge-vector *Aedes rossicus* was only found infected with SINV in 2002. This may indicate that in years with increased SINV activity in the enzootic cycle, virus is disseminated to mosquito species not normally involved in SINV circulation. Similar observations from South Africa, where both SINV and WNV are endemic, provide annual detection in the enzootic vector *Culex univittatus*, and in additional species during a year with large number of human cases [5]. For outbreak risk evaluation, we see no need for including *Aedes rossicus* in the SINV assay.

Climate variables can be used as risk indicators for WNV and SINV outbreaks in South Africa [5, 6, 39], and is therefore a potential indicator of SINV outbreaks in northern Europe. The transmission of SINV from the natural enzootic cycle to humans depend on the bridge-vector *Aedes cinereus* [8, 15]. Ecologically, *Aedes cinereus* is a floodwater mosquito, and floodwater mosquito occurrence and abundance are directly dependent on inundation and floods [29, 40, 41]. Interestingly, floodwater mosquitoes including *Aedes cinereus* are potential vectors of the bacteria *Francisella tularensisholarctica* causing Tularemia [42, 43], and increased floodwater mosquito prevalence in late summer has been coupled to outbreaks of Tularemia in Sweden [44]. Thus, climate variables are potential indicator of SINV induced polyarthritis outbreaks in northern Europe.

The phylogenetic analyses support the current view of SINV having a monophyletic history [2]. Although the last common ancestor of the currently acknowledged five SINV genotypes suggest an evolution over roughly the last 2000 years, SINV-1 strains have been introduced to Sweden and Finland no longer than a century ago. They slowly emerged in the 1970s which led to the recognition of SINV-caused Ockelbo disease in Sweden and Pogosta disease in Finland in the late 1960s and early 1970s [4, 20]. A total of currently 4 clades have formed which suggests for four independent introductions into Scandinavia. Alternatively, a single introduction followed by a further spread, most likely via birds, may have formed the four clades. However, the SINV strains from Finland intermingling with two of the four clades argue for distinct introduction events [23]. Further studies based on full genome sequencing of a large number of Swedish SINV-I isolates will be useful for a more comprehensive evaluation of the hypothesis of local long-term maintenance of SINV in its northernmost occurrence.

Long term local maintenance of SINV-I requires annual transmission and amplification of the virus in the enzootic transmission cycle involving the main enzootic vector *Culex torrentium* and the main vertebrate amplifying hosts of the genus *Turdus* [8]. Our results suggest that increased SINV-I prevalence in the bridge-vector *Aedes cinereus* and in the enzootic vectors *Culex torrentium/pipiens* constitute valuable outbreak markers. Our results also revealed that surveillance of virus activity should focus on vector mosquito sampling during middle of July until September. Further, the cyclic increase in virus prevalence was shown neither to be related to any reintroduction of SINV exotic strains (this study and [23]), nor to variation in bird host immunity [38]. The continued evaluation of the seven-year cycle of SINV polyarthritis outbreaks should examine the annual virus prevalence patterns over more triads of pre-outbreak, outbreak and post-outbreak years, preferable including explanatory variables such as climate.

## Supporting information

S1 Supporting Information The mosquito species collected during early, late and full season, in the year 2000 mosquito diversity study in the River Dalälven floodplains, central Sweden (according to Schäfer et al. 2008), and tested for virus by cell culture.
(DOCX)

S2 Supporting Information Maximum likelihood tree derived from a ClustalW alignment with partial Sindbis virus sequences of strains from Sweden, Finland, Germany, Egypt, Azerbaijan, Australia, India and New Zealand. Bootstrapping was performed with 1000 replicates and percent values above 80% are shown.
(TIF)
