# Supplementary material for: Sindbis virus polyarthritis outbreak signalled by virus prevalence in the mosquito vectors
Source: PLoS Negl Trop Dis. 2019 Aug 29;13(8):e0007702. doi: 10.1371/journal.pntd.0007702 (PMC6738656; doi:10.1371/journal.pntd.0007702)
Supplement: S1 Supporting Information — The mosquito species collected during early, late and full season, in the year 2000 mosquito diversity study in the River Dalälven floodplains, central Sweden (according to Schäfer et al. 2008), and tested for virus by cell culture. (DOCX) [file pntd.0007702.s001.docx]

S1. The mosquito species collected during early, late and full season, in the year 2000 mosquito diversity study in the River Dalälven floodplains, central Sweden [28], and tested for virus by cell culture.

| **Species** | **Early season**^a^ | **Late season** | **Full season** |
| --- | --- | --- | --- |
| *Aedes annulipes* | 0/55 (0.0)^b^ | 0/16 (0.0) | 0/71 (0.0) |
| *Aedes cantans* | 0/1044 (0.0) | 0/92 (0.0) | 0/1136 (0.0) |
| *Aedes cataphylla* | 0/1 (0.0) | 0/0 (0.0) | 0/1 (0.0) |
| *Aedes cinereus* | 0/2568 (0.0) | 1/11,184 (0.09) | 1/13,752 (0.07) |
| *Aedes communis* | 0/1286 (0.0) | 0/221 (0.0) | 0/1507 (0.0) |
| *Aedes diantaeus* | 0/9 (0.0) | 0/0 (0.0) | 0/9 (0.0) |
| *Aedes excrucians* | 0/2 (0.0) | 0/0 (0.0) | 0/2 (0.0) |
| *Aedes intrudens* | 0/2281 (0.0) | 0/92 (0.0) | 0/2373 (0.0) |
| *Aedes punctor* | 0/528 (0.0) | 0/612 (0.0) | 0/1140 (0.0) |
| *Aedes rossicus* | 0/3570 (0.0) | 0/16,985 (0.0) | 0/20,555 (0.0) |
| *Aedes sticticus* | 0/12,490 (0.0) | 0/30,783 (0.0) | 0/43,273 (0.0) |
| *Aedes vexans* | 0/0 (0.0) | 0/122 (0.0) | 0/122 (0.0) |
| *Anopheles claviger* | 0/1 (0.0) | 0/4 (0.0) | 0/5 (0.0) |
| *Anopheles maculipennis* | 0/29 (0.0) | 0/1 (0.0) | 0/30 (0.0) |
| *Coquilletidia richiardii* | 0/204 (0.0) | 0/12 (0.0) | 0/216 (0.0) |
| *Culex torrentium/pipiens* | 0/22 (0.0) | 0/4 (0.0) | 0/26 (0.0) |
| *Culiseta alaskaensis* | 0/147 (0.0) | 0/1 (0.0) | 0/148 (0.0) |
| *Culiseta bergrothi* | 0/0 (0.0) | 0/1 (0.0) | 0/1 (0.0) |
| *Culiseta morsitans* | 0/37 (0.0) | 0/125 (0.0) | 0/162 (0.0) |
| *Culiseta ochroptera* | 0/1 (0.0) | 0/0 (0.0) | 0/1 (0.0) |
| Total | 0/24,275 (0.0) | 1/60,255 (0.02) | 1/84,530 (0.01) |

^a^ Early season is week 21-28, late season is week 33-38, and full season is week 21-38.

^b^ Number of virus isolates/number of mosquitoes assayed (Infection Rate/1000 mosquitoes).
