# Supplementary figures and images for: Sindbis virus polyarthritis outbreak signalled by virus prevalence in the mosquito vectors
Source: PLoS Negl Trop Dis. 2019 Aug 29;13(8):e0007702. doi: 10.1371/journal.pntd.0007702 (PMC6738656; doi:10.1371/journal.pntd.0007702)

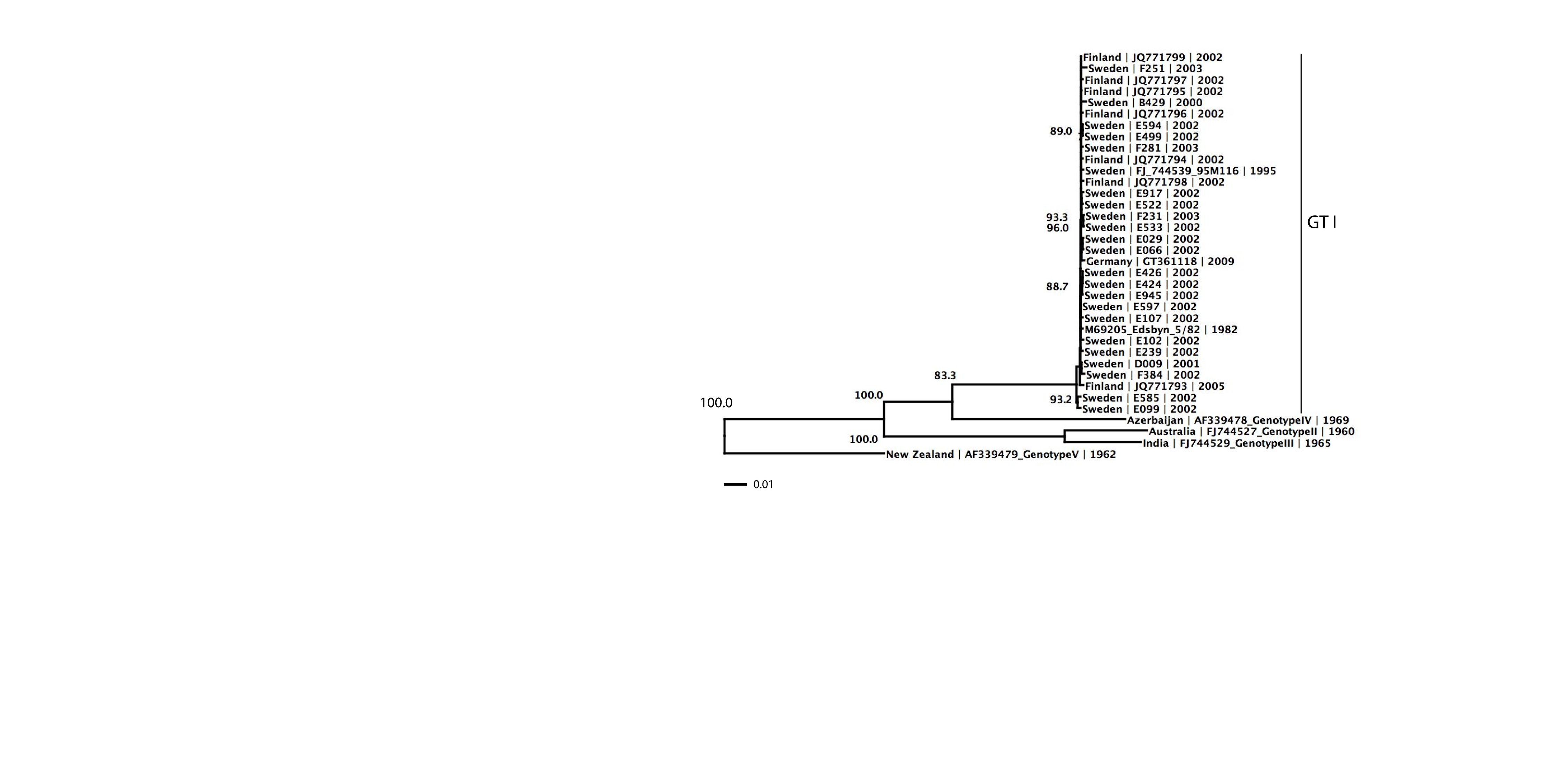

Supplement: S2 Supporting Information — Maximum likelihood tree derived from a ClustalW alignment with partial Sindbis virus sequences of strains from Sweden, Finland, Germany, Egypt, Azerbaijan, Australia, India and New Zealand. Bootstrapping was performed with 1000 replicates and percent values above 80% are shown. (TIF) [file pntd.0007702.s002.tif]
